# Multiple Origins of Knockdown Resistance Mutations in the Afrotropical Mosquito Vector *Anopheles gambiae*

**DOI:** 10.1371/journal.pone.0001243

**Published:** 2007-11-28

**Authors:** João Pinto, Amy Lynd, José L. Vicente, Federica Santolamazza, Nadine P. Randle, Gabriele Gentile, Marta Moreno, Frédéric Simard, Jacques Derek Charlwood, Virgílio E. do Rosário, Adalgisa Caccone, Alessandra della Torre, Martin J. Donnelly

**Affiliations:** 1 Centro de Malária e outras Doenças Tropicais, Instituto de Higiene e Medicina Tropical, Lisbon, Portugal; 2 Vector Group, Liverpool School of Tropical Medicine, Liverpool, United Kingdom; 3 Istituto Pasteur-Fondazione Cenci-Bolognetti, Sezione di Parassitologia, Dipartimento di Scienze di Sanità Pubblica, Università di Roma - La Sapienza, Rome, Italy; 4 Dipartimento di Biologia, Università di Roma - Tor Vergata, Rome, Italy; 5 Centro Nacional de Medicina Tropical, Instituto de Salud Carlos III, Madrid, Spain; 6 Organisation de Coordination pour la lutte contre les Endemies en Afrique Centrale, Yaoundé, Cameroon; 7 Institut de Recherche pour le Développement, Yaoundé, Cameroon; 8 DBL – Institute for Health Research and Development, Charlottenlund, Denmark; 9 Yale Institute for Biospheric Studies and the Department of Ecology and Evolutionary Biology, Yale University, New Haven, Connecticut, United States of America; Centre for DNA Fingerprinting and Diagnostics, India

## Abstract

How often insecticide resistance mutations arise in natural insect populations is a fundamental question for understanding the evolution of resistance and also for modeling its spread. Moreover, the development of resistance is regarded as a favored model to study the molecular evolution of adaptive traits. In the malaria vector *Anopheles gambiae* two point mutations (L1014F and L1014S) in the voltage-gated sodium channel gene, that confer knockdown resistance (*kdr*) to DDT and pyrethroid insecticides, have been described. In order to determine whether resistance alleles result from single or multiple mutation events, genotyping of the *kdr* locus and partial sequencing of the upstream intron-1 was performed on a total of 288 *A. gambiae* S-form collected from 28 localities in 15 countries. Knockdown resistance alleles were found to be widespread in West Africa with co-occurrence of both 1014S and 1014F in West-Central localities. Differences in intron-1 haplotype composition suggest that *kdr* alleles may have arisen from at least four independent mutation events. Neutrality tests provided evidence for a selective sweep acting on this genomic region, particularly in West Africa. The frequency and distribution of these *kdr* haplotypes varied geographically, being influenced by an interplay between different mutational occurrences, gene flow and local selection. This has important practical implications for the management and sustainability of malaria vector control programs.

## Introduction

The development of insecticide resistance is regarded as a favored empirical model to study the molecular evolution of adaptive traits. The onset of resistance is a relatively fast and well-documented event in many insect species, particularly those of medical and economic interest. In addition, the resistance phenotype is usually associated with a few major genes or gene classes and mutations therein that confer the trait [1]. Within this framework, particular attention has been given to determining how often resistance mutations arise in natural populations. This is a fundamental question for understanding the evolution of resistance and also for modeling its spread.

In *Culex pipiens*, there is evidence for a single origin of a duplication in esterase genes, conferring metabolic resistance to organophosphates, followed by global spread through migration [2]. A single origin and global dispersal of a P450 allele associated with resistance to DDT was also described in *Drosophila melanogaster* [3]. Multiple origins of resistance alleles involving different genes have also been documented in several insect species. These include point mutations at the voltage-gated sodium channel gene of *Bemisia tabaci* and *Myzus persicae* [4], [5], γ-aminobutyric acid (GABA) receptors in *Tribolium castaneum* [6] and esterase genes in *Lucilia cuprina* [1].

In *Anopheles gambiae sensu stricto*, the principal Afrotropical malaria vector, two point mutations at the voltage-gated sodium channel gene confer knockdown resistance (*kdr*) to DDT and pyrethroid insecticides. Martinez-Torres et al. [7] identified a Leucine-Phenylalanine substitution at position 1014 (L1014F) of the gene encoding the S6 transmembrane segment of domain II of the sodium channel, in laboratory strains derived from field resistant samples of Burkina Faso and Ivory Coast. A second mutation, a Leucine-Serine substitution at the same codon (L1014S), has been identified in a colony derived from specimens from Kenya [8].

Field surveys revealed a widespread distribution of the 1014F allele in West Africa [9], [10]. In addition, significant differences were found in the frequency of this allele between two molecular forms, denoted M and S, that are considered units of incipient speciation within *A. gambiae* [11]. These forms are characterized by sequence differences in transcribed and non-transcribed spacers of the ribosomal DNA. The S-form is the most widespread throughout Sub-Saharan Africa while the M-form is mostly confined to the West part of Africa, from Senegal to Angola, with extensive overlapping distribution with the S-form. In general, the 1014F allele is common in the S-form but rare in the M-form, even when populations of both forms occur in sympatry [11], [12]. In the few M-form populations where it has been found, sequencing analysis of the upstream intron-1 of the *kdr* locus showed that the 1014F allele apparently occurred through introgression with the S-form [13]. Less information is available on the distribution of the 1014S allele. It appears to be less widespread, occurring mainly within East Africa [14]. However, recent surveys have reported the co-occurrence of both 1014F and 1014S alleles in localities of Gabon, Cameroon and Uganda [15]–[17].

The distribution and frequency of these mutations poses serious questions about the sustainability of insecticide-based vector control programs. This is particularly evident when one considers that pyrethroids are the only insecticides recommended by the World Health Organization for insecticide-treated materials and that DDT is being re-introduced for malaria control in several countries [18]. Knowledge of the way *kdr* resistance is evolving in *A. gambiae* is therefore of great epidemiological importance. Whether these mutations have arisen only once and are spreading throughout the species distribution or if multiple independent mutation events have occurred, remains to be understood. Also, it is central for control purposes to evaluate the role of local selection pressures and of migration in shaping the distribution and frequency of *kdr* alleles. This would allow to design more finely tuned control strategies that take into account current and historical selection pressures and gene flow patterns.

In this study, we have genotyped the *kdr* locus and sequenced the upstream intron-1 in samples of *A. gambiae* S-form throughout Sub-Saharan Africa in order to, i) establish a minimum number of mutation events giving rise to *kdr* alleles, ii) characterize heterogeneities in the geographic distribution of *kdr* alleles, and iii) relate these to aspects of selection and patterns of population structure known for this species.

## Materials and Methods

### Samples

**Figure 1:**
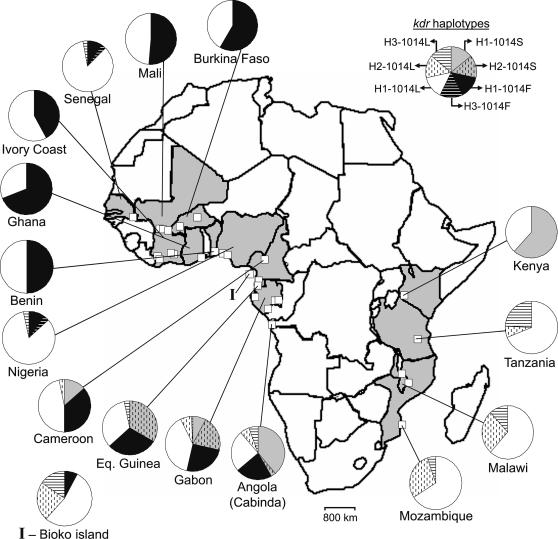
Geographic distribution of *kdr* alleles and most common intron-1 haplotypes of *A. gambiae*. Gray-shaded countries are those included in the study. White squares in the map are the approximate locations of collection sites. Pie charts show the relative frequencies of *kdr* haplotypes (*i.e. kdr* alleles and corresponding intron-1 haplotypes), per country. Labels for each *kdr* haplotype are shown in the example pie chart, at the upper-left corner of the figure.

DNA samples from individual females identified by PCR as *A. gambiae s.s.* S-form [19], were obtained from 28 collection sites in 15 African countries (Figure 1, see also Table S1 in Supporting Information). Except for the sample from Cameroon that was composed by adults that emerged from field collected larvae, all the other samples were composed by field collected adults. Due to sampling constraints inherent to the fact that *kdr* alleles are rarely found in the M-form, this study dealt only with samples of *A. gambiae* S-form.

### Genotyping and sequencing procedures

The *kdr* locus was genotyped either by allele-specific-PCR (AS-PCR) or Hot Oligonucleotide Ligation Assay (HOLA) methods [7], [8], [20]. Genotypes were confirmed in a subset of samples by direct sequencing of a 293 bp (base pairs) fragment containing the *kdr* locus and the downstream intron-2, using primers Agd1 and Agd2 [7], [16]. Direct sequencing of the intron-1 was performed according to previously described protocols [10], [13]. Sequences from both strands were obtained for each specimen, aligned in BIOEDIT v. 7.0.5.2. [21] and corrected manually. Part of the sequences have been produced in a previous study [10] (Table S1).

In order to recover full haplotypes comprising both intron-1 sequences and corresponding *kdr* alleles from heterozygotes at more than one site, a Bayesian approach based on *a priori* predictions from the coalescent theory was used to reconstruct haplotypes from population genotypic data, implemented by the software Phase 2.1. [22]. Predicted haplotypes were confirmed by cloning of a fragment of 568 bp containing the *kdr* locus and all segregating sites at intron-1 in a subset of individuals. Initial fragment amplification was carried out using primers kdrCL-F (AAATGTCTCGCCCAAATCAG) and kdrCL-R (GCACCTGCAAAACAATGTCA), located upstream at positions 602–621 in the intron-1 and downstream at positions 1150–1169 at the end of the intron-2, respectively (nucleotide positions as in [13]). The PCR mixture contained 1× PCR Buffer (Promega, Madison WI, USA), 2 mM of MgCl_2_, 200 µM of a dNTPs equimolar mix, 1 U *Taq* DNA polymerase (Promega, Madison WI, USA) and 0.25 µM of each primer, in a total volume of 50 µl. Cycling conditions were 94°C for 5 min, followed by 35 cycles each with 94°C for 30 sec, 50°C for 35 sec and 72°C for 60 sec, followed by a final extension step of 10 min at 72°C. Amplified products were cloned into pCR 2.1. TOPO TA vectors (Invitrogen, Carlsbad CA, USA). In order to obtain both haplotypes, three to five clones were sequenced for each individual.

### Data analysis

Estimates of DNA polymorphism at the intron-1, including the number of segregating sites, number of haplotypes, haplotype diversity and nucleotide diversity, were obtained using DnaSP v. 4.10.9 [23]. The same software was used to perform neutrality tests in order to infer if selection is acting upon the analyzed intron-1 region. Tajima's *D* test [24] compares two estimates of θ ( = 4*N_e_*μ for diploid organisms, where *N_e_* is the effective population size and μ the mutation rate), one based on the number of segregating sites and the other on the average number of pairwise nucleotide differences, that should be equal under the neutral mutation model. If selection is affecting the genomic region, estimates will differ, as selection will affect more readily the number segregating sites. Fu and Li's D* and F* tests [25] compare two estimates of θ, based on the number of mutations found in internal and external branches of the genealogy, respectively. In the presence of purifying selection, an excess of mutations in external branches is likely to occur as deleterious mutations are maintained at low frequencies. However, if balancing selection occurs this may result in a deficit of external mutations. Fu [26] proposed the *F_S_* statistic, which is based on expectations of haplotype frequency distribution for a given value of θ derived from the average number of pairwise nucleotide differences. When there is an excess of recent mutations, θ estimated by the mean number of pairwise nucleotide differences will tend to be smaller than that based on the number of alleles. Negative values of *F_S_* are expected as an indication of genetic hitchhiking or population growth [26].

Genealogical relations among haplotypes were estimated by constructing a parsimony network using the TCS software [27], which uses the parsimony algorithm of Templeton et al. [28] to perform a pairwise calculation of the number of mutational steps between haplotypes until a probability threshold of 95% is exceeded. To conduct this analysis, full haplotypes comprising intron-1 and the *kdr* locus retrieved from both cloning and gametic phase analysis by Phase 2.1. were used.

In order to infer if recombination between the *kdr* locus and polymorphic positions in the intron-1 could be a more likely cause for the origin of the observed haplotypes, two estimates of recombination rate were obtained. The population background recombination rate ρ (ρ = 4*N_e_r*, where *r* is the rate of crossing over per base pair) was estimated by the method proposed by Li and Stephens [29], implemented in Phase 2.1. In this method, each haplotype is reconstructed as a mosaic of previously considered haplotypes and ρ is estimated from the average length of the mosaic pieces (see also [30]). A median value of ρ was obtained from 100 randomly selected sequences and bootstrapped 95% confidence intervals were obtained for the estimates (10,000 replicates). The minimum number of recombination events, *R_m_*, in the history of a sample was estimated by the “four-gamete test” method described by Hudson and Kaplan [31], as implemented in DnaSP.

## Results

A total of 288 *A. gambiae* S-form were analyzed (2*N* = 576 sequences), with a mean of 10 individuals per locality. Knockdown resistance associated alleles were not homogeneously present in the samples analysed (Figure 1; Table S1): i) in Western African samples (2*N* = 202 sequences), from Nigeria to Senegal, the L1014F was the only mutation found; ii) in the West-Central region of Africa (2*N* = 254), comprising Angola, Gabon, Equatorial Guinea and Cameroon, both *kdr* alleles co-occurred in 8 localities surveyed. In this region, the 1014S allele was absent only in the sample of Bioko island (2*N* = 14); iii) in East Africa (2*N* = 120), the L1014S mutation was found only in the Kenyan sample (2*N* = 26) and no 1014F alleles were found. Overall, *kdr* alleles were present in 23 out of the 28 localities sampled. Direct sequencing of the *kdr* locus in 137 individuals confirmed the genotypes obtained either by HOLA or AS-PCR. No additional polymorphisms were detected.

**Figure 2:**
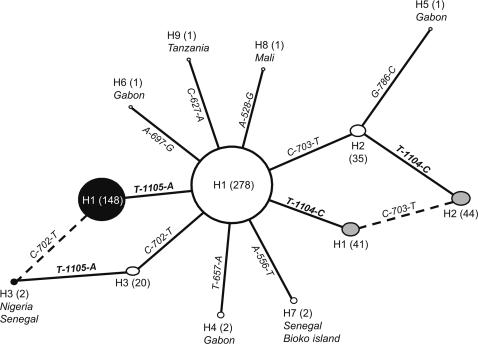
TCS network showing the genealogical relations among *kdr* haplotypes of *A. gambiae*. Each haplotype is represented by a circle with a size proportional to its frequency in the sample (in parenthesis, 2*N* = 576). Countries where the most rare haplotypes (*i.e.* frequency <0.5%) were found are also reported. Mutational steps are represented by lines with the indication of the mutation from the immediate ancestral haplotype (*kdr* mutations in bold). Dashed lines represent reticulation ambiguities (see Discussion). White circles: haplotypes carrying the wild-type 1014L allele. Gray circles: haplotypes carrying the 1014S allele at the *kdr* locus (T-1104-C). Black circles: haplotypes carrying the 1014F allele at the *kdr* locus (A-1105-T).

Sequencing analysis of a 438 bp region of intron-1 revealed 8 polymorphic sites of which four were singletons (positions 528, 627, 697 and 786, as in [13]). No insertions or deletions were found. A total of 9 different intron-1 haplotypes were detected (Genebank accession n° EU078886–EU078894). Of these, there were three predominant haplotypes resulting from single step mutations at positions 702 (T/C) and 703 (C/T). Haplotype H1 (702T-703C, formerly S1 in [10]) was the most frequent and widespread (81.1% of all 576 sequences). In the West African region, it was fixed in localities from Benin, Ghana, Ivory Coast and Burkina Faso (Figure 1). It was also the only haplotype found in Asembo, Kenya, which was the only East African sample that had the 1014S allele. Haplotype H2 (702T-703T, formerly S6 in [10]) was found in both West-Central (23.2%, 2*N* = 254) and East Africa (16.7%, 2*N* = 120), but it was absent in West African sites. Haplotype H3 (702C-703C, formerly M1 in [10]), also had an extensive but patchier distribution. This haplotype occurred at a higher frequency in East Africa (10.0%, 2*N* = 120) than in West-Central (2.4%, 2*N* = 254) and Western (2.0%, 2*N* = 202) regions. The remaining 6 haplotypes were found at very low frequencies (<0.5%, 2*N* = 576) representing mostly local variants (Figure 2).

Geographical differences were also evident in the estimates of genetic diversity (Table S1). In Western African sites variation at the intron-1 was lowest, with an overall haplotype and nucleotide diversity of 0.058 (SD: ±0.023) and nucleotide diversity of 0.00013 (SD: ±0.00005). West-Central African and East African sites showed similar levels of diversity, with overall haplotype diversity of 0.422 (SD: ±0.030) and 0.440 (SD: ±0.048), and nucleotide diversity of 0.00102 (SD: ±0.00008) and 0.00232 (SD: ±0.00014), respectively.

Given the above mentioned differences, neutrality tests were performed on three groups of samples that were pooled according to the West, West-Central and East African regions, rather than on single localities (Table 1). Significant departures from neutrality were detected by *F_s_* both in the West and West-Central regions, but with a larger negative value for the West region. All other tests were non-significant, with the exception of the F* value obtained also for the West region. In the East region, values obtained for all tests were closest to the expectations of neutrality.

**Table 1:** Neutrality tests according to geographic region.

Region^a^	*N* b	*S* (*η_s_*) c	*K* d	*D*	*D* *	*F* *	*F*_S_
West	202	3 (2)	0.059	−1.416^ns^	−2.241^ns^	−2.333*	−5.376*
West-Central	254	6 (3)	0.448	−1.071^ns^	−2.138^ns^	−2.111^ns^	−3.327*
East	120	3 (1)	0.478	−0.253^ns^	−0.627^ns^	−0.597^ns^	−0.399^ns^

a West: Nigeria, Benin, Ghana, Ivory Coast, Burkina Faso, Mali and Senegal. West-Central: Angola, Gabon, Equatorial Guinea (including Bioko island) and Cameroon. East: Tanzania, Kenya, Malawi and Mozambique.

b Sample size (n° of chromosomes).

c Number of segregating sites, *S*, and number of singleton mutations (η*_s_*).

d Average number of pairwise nucleotide differences. Neutrality tests: Tajima's *D* test [24], Fu and Li D* and F* tests [25], Fu *F*_S_ test [26].

ns : non-significant.

* : *P*<0.05.

Sequencing of cloned fragments containing the *kdr* locus and intron-1 polymorphisms from 14 individuals confirmed the haplotypes predicted by the gametic phase analysis using Phase 2.1. software. Errors of misincorporation of nucleotides were estimated as 0.0017 per base pair, by comparing the cloned sequences with those obtained by direct sequencing of PCR products. In addition, three haplotypes, instead of the expected two, were seen in 3 individuals. This may have been due to the presence of male DNA in the female's spermatheca or to *in vitro* recombination by jumping PCR [32].

By reconstructing individual full haplotypes from intron-1 sequences and correspondent *kdr* genotypes, it was possible to distinguish four different *kdr* haplotypes (Genebank accession n° EU078895–EU078898). Their genealogical relations are illustrated in the TCS network in Figure 2. The network suggests four independent mutation events giving rise to *kdr* haplotypes. Haplotypes H1-1014F and H1-1014S derive from single mutational steps from the common ancestor H1-1014L. Haplotypes H2-1014S and H3-1014F are the result of two mutational steps, but ambiguous connections were found for these tip haplotypes (Figure 2). The geographic distribution of *kdr* haplotypes is shown in Figure 1. The West Central African region presented the highest diversity, with H1-1014F, H1-1014S and H2-1014S co-occurring in sites from Gabon and northern Angola.

The estimate of background recombination rate, ρ, was 8.01×10^−7^ (Bootstrap 95% CI: 1.64×10^−7^–2.21×10^−6^). In order to account for recombination only between the *kdr* locus and the segregating sites in intron-1 discriminating the different *kdr* haplotypes, analysis was repeated using only positions 702 and 703 in intron-1 and the *kdr* locus, giving an estimate of *ρ* = 8.13×10^−5^ (Bootstrap 95%CI: 2.36×10^−5^–1.84×10^−4^).

The minimum number of recombination events in the history of the sample was estimated as *R_m_* = 1, corresponding to a single recombination event between polymorphic positions 703 (yielding intron-1 haplotypes H1 and H2) and 1104 (*i.e.* the *kdr* mutation L1014S).

## Discussion

Analysis of the upstream intron-1 of the *kdr* locus suggests at least four independent origins of *kdr* alleles in the principal Afrotropical malaria vector *A. gambiae* S-form. Two of these events are unequivocal since they result from single-step mutations at the *kdr* locus from a common progenitor haplotype (H1) resulting in two different phenotypes, *i.e.* the previously described aminoacidic substitutions L1014F and L1014S [7], [8]. The origins of the other two *kdr* haplotypes are not as clear-cut, given the reticulations obtained in the TCS network. Following the guidelines of Templeton et al. [23] and Crandall and Templeton [33] to resolve network ambiguities, it could be hypothesized that these *kdr* haplotypes have arisen from mutations at the intron-1 in ancestors already carrying the *kdr* mutation. However, these guidelines might not be appropriate in this case, since they are based on a neutral model, in which the most frequent haplotypes are the oldest and thus more likely to give rise to tip haplotypes. These predictions do not hold when neutrality is violated, as it is probably the case of this dataset. Selection through insecticide pressure will favor *kdr* alleles and remove wild-type alleles. Therefore, the frequency of haplotypes carrying the wild-type allele will be lower than expected under neutrality, and so frequency need not reflect age. In this case, haplotypes H2-1014S and H3-1014 could therefore have been generated by two additional mutation events at the *kdr* locus. It is also important to note that given the low genetic variation found at the intron-1, the number of independent mutations detected may still be an underestimate of the actual number of mutation events that have occurred at the *kdr* locus of *A. gambiae*.

Two-step *kdr* haplotypes may also have arisen by recombination between an intron-1 haplotype carrying a *kdr* allele and a different haplotype carrying the wild-type allele. Indeed, Hudson and Kaplan's [31] *R_m_* predicted one recombination event that could have given rise to H1-1014S and H2-1014S haplotypes. However, the voltage-gated sodium channel gene maps in Division 20C on the centromeric end of arm L of chromosome-2 [8], and recombination tends to be reduced in regions surrounding the centromere. In addition, although an earlier origin cannot be fully excluded, mutations originating resistance alleles are more likely to have arisen with the onset of selective pressures from insecticide use. This assumption is supported by the observation that a number of insect species share exactly the same resistance-associated polymorphisms, a situation unlikely to be met in the absence of insecticide selection [5], [6]. Selective pressures at the sodium channel of *A. gambiae* should thus be coincident with the introduction of DDT in Africa in the mid-1940's for both agricultural and vector control purposes [18], [34]. Assuming 12–24 generations per year for *A. gambiae* [35], this would imply about 700 to 1400 generations for recombination to have occurred. Taking 10^−4^ as the upper confidence limit of the highest estimate of ρ obtained, one would expect a recombination event every *ca.* 10,000 generations, or 416 years assuming the overestimate of 24 generations per year for *A. gambiae*. This time-window is probably not enough for recombination to have generated the observed *kdr* haplotypes.

The H1-1014F *kdr* haplotype was the most widespread throughout West and West-Central Africa. This extensive distribution together with the high frequencies found in West African sites, that also showed limited intron-1 diversity, suggest that dispersal through migration followed by local selection have shaped the distribution and frequency of this *kdr* haplotype. It is worth noting that the 1014F allele was also detected in East Africa (Uganda), but no information on the associated intron-1 haplotype is available [17]. The more limited distribution of the two *kdr* haplotypes carrying the 1014S allele raises the possibility of a more recent origin of this allele in West-Central Africa from at least two independent mutation events. However, while haplotype H2-1014S is confined to the West-Central African region, haplotype H1-1014S was also found in Kenya. Earlier studies indicated that only a few major physical barriers limit gene flow between *A. gambiae* populations [36]. However, genetic discontinuities within molecular forms have recently been detected in West Africa, associated with specific chromosomal arrangements or different ecological zones [37]–[39]. Given the low resolution (*i.e*. low polymorphism) of the intron-1 region analyzed and the unavailability of samples from intermediate Central African localities for this study, the possibility of independent mutations giving rise to East and West African H1-1014S haplotypes cannot be ruled out.

The H3-1014F haplotype was detected in only two individuals from Senegal and Nigeria, homozygous for the intron-1 H3 haplotype and 1014L/1014F heterozygous at the *kdr* locus. Interestingly, in West Africa, haplotype H3 was shown to be almost exclusive of the M-form [10]. The few M-form samples available from the sites surveyed in this study did not show any *kdr* alleles (data not shown), which precluded the analysis of *kdr* haplotypes in *A. gambiae* M-form. However, the occurrence of additional independent *kdr* mutation events in the M-form has been recently suggested for Bioko island [40] and further studies on this form are certainly needed.

Low genetic variation was observed in the intron-1 of *A. gambiae* when compared to other insect species that are also subjected to insecticide pressure [4], [5]. The overall low genetic diversity in the intron-1 could reflect a “centromere effect” [41]. In addition, such low variation may be a consequence of a recent selective sweep [10], [13]. A selective sweep occurs when an allele rapidly increases its frequency due to positive selection. Through genetic hitchhiking, the frequency of linked alleles in the flanking regions of the locus under selection can also increase, thus reducing genetic variation. Evidence of a selective sweep comes from the neutrality tests. The highest departure from neutrality was detected by the *F_S_* statistic, in the West African region. In a comparison of the tests used, *F_S_* showed the highest power in detecting departures from neutrality under a genetic hitchhiking model [26]. The values closer to neutrality obtained for the other tests may thus indicate that selection is not acting directly on the intronic region, but through genetic hitchhiking. The highest *F_S_* estimated for the West African region may be a consequence of stronger selective pressures due to increased insecticide use in this region. For the past 20 years, cotton production has increased remarkably in the West African region, making it the world's third largest producer [42]. This increase was most likely followed by an increased use of pyrethroid insecticides. Selection of *kdr* resistance in *A. gambiae* in cotton areas associated with the use of pyrethroids has been well documented [43].

No departures from neutrality were detected in East Africa, where only the 1014S allele was found at a single locality (Asembo, Kenya). Stump et al. [14] have shown that the presence of the 1014S allele pre-dates the use of pyrethroids in Asembo and report an increase in the frequency of this allele with the onset of a large scale permethrin-treated bednets trial. These findings coupled with the presence of a single intron-1 haplotype in this locality also suggest that a local selective sweep of the 1014S allele may have recently occurred in this area.

The geographic distribution of *kdr* haplotypes should reflect the interplay between the evolutionary forces of mutation, gene flow and selection. In *A. gambiae* S-form there is evidence suggesting that at least four mutation events have originated *kdr* alleles. These insecticide resistance associated alleles are widespread and reach high frequency, especially in West and West-Central Africa. Of particular relevance is the co-occurrence of both 1014F and 1014S alleles in the West-Central region, not only at the population but also at the individual level (*i.e.* 1014S/1014F heterozygotes). The phenotypic outcome of these genotypes in terms of individual response to insecticides remains to be uncovered. More ecological studies are needed, relating levels of insecticide resistance with the genotypic composition at the *kdr* locus and with the analysis of other resistance mechanisms (*e.g.* metabolic, behavioral), particularly for the West-Central African region. This would not only provide further insights into the molecular evolution of insecticide resistance, but would also have important practical implications for vector control. It remains to be fully clarified the extent of the contribution of *kdr* mutations to the resistance phenotype, particularly in cases where non-target site resistance mechanisms are also present [8]. This information is essential for making use of *kdr* frequency variation as a measuring tool in insecticide resistance monitoring systems. A clear definition of the role of different resistance mechanisms is therefore central to evaluate the impact of insecticide-based vector control programs aimed at lowering the malaria burden, the major health problem in developing countries from tropical regions.

## Supporting Information

Table S1 Collection sites, sample sizes, *kdr* allele frequencies and estimates of DNA polymorphism at the intron-1
(0.11 MB DOC)
