# Supplementary material for: Multiple Origins of Knockdown Resistance Mutations in the Afrotropical Mosquito Vector Anopheles gambiae
Source: PLoS One. 2007 Nov 28;2(11):e1243. doi: 10.1371/journal.pone.0001243 (PMC2080755; doi:10.1371/journal.pone.0001243)
Supplement: Table S1 — Collection sites, sample sizes, kdr allele frequencies and estimates of DNA polymorphism at the intron-1 (0.11 MB DOC) [file pone.0001243.s001.doc]

**Supporting information**

**Table S1. Collection sites, sample sizes, *kdr*** allele frequencies and estimates of DNA polymorphism at the intron-1

| Country | Locality (year of collection)  Latitude/Longitude | *N* ^a^ | *kdr* alleles ^b^ (%) | | | Intron-1 polymorphism | | | |
| --- | --- | --- | --- | --- | --- | --- | --- | --- | --- |
|  |  |  | 1014 L | 1014S | 1014F | *S* ^c^ | *h* ^d^ | *Hd* ^e^ | *π* ^f^ |
|  | | | | | | | | | |
| **West African region** | | | | | | | | | |
| Senegal | Kedougou* (2002)  12°36'N, 12°14'W | 17 | 88.2 | 0.0 | 11.8 | 2 | 3 | 0.169 | 0.00039 |
| Mali | Total | 19 | 50.0 | 0.0 | 50.0 | 1 | 2 | 0.052 | 0.00012 |
|  | N'Gabacoro* (2000)  12°41'N, 07°50'W | 8 | 75.0 | 0.0 | 25.0 |  |  |  |  |
|  | Bananbani* (2000)  12°48'N, 08°03'W | 7 | 28.6 | 0.0 | 71.4 |  |  |  |  |
|  | Pimperena* (2000)  11°28'N, 05°42'W | 4 | 37.5 | 0.0 | 62.5 |  |  |  |  |
| Ivory Coast | Total | 13 | 57.7 | 0.0 | 42.3 | 0 | 1 | - | - |
|  | Danta* (1998)  07°01'N, 08°09'W | 2 | 100.0 | 0.0 | 0.0 |  |  |  |  |
|  | M’bé* (1998)  07º14'N, 05º01'W | 3 | 33.3 | 0.0 | 66.7 |  |  |  |  |
|  | Yakoffikro (1998)  07°55'N, 05°37'W | 3 | 0.0 | 0.0 | 100.0 |  |  |  |  |
|  | Ziglo* (1998)  06°33'N, 07°47'W | 5 | 90.0 | 0.0 | 10.0 |  |  |  |  |
| Burkina  Faso | Total | 6 | 41.7 | 0.0 | 58.3 | 0 | 1 | - | - |
|  | Dioulassouba* (2001)  11°02'N, 04°13’W | 3 | 0.0 | 0.0 | 100.0 |  |  |  |  |
|  | Goundry (1998)  12°35'N, 01°02'W | 3 | 83.3 | 0.0 | 16.7 |  |  |  |  |
| Ghana | Accra (2002)  05°38'N, 00°15'E | 26 | 30.8 | 0.0 | 69.2 | 0 | 1 | - | - |
| Benin | Bohicon* (1998)  07°10'N, 02°05'E | 5 | 50.0 | 0.0 | 50.0 | 0 | 1 | - | - |
| Nigeria | Total | 15 | 86.7 | 0.0 | 13.3 | 1 | 2 | 0.128 | 0.00029 |
|  | Kobape* (2001)  07°00'N, 03°00'E | 7 | 92.9 | 0.0 | 7.1 |  |  |  |  |
|  | Olugbo* (2001)  07°20'N, 03°30'E | 8 | 81.3 | 0.0 | 18.8 |  |  |  |  |
|  | | | | | | | | | |
| **West-Central African region** | | | | | | | | | |
| Cameroon | Magba  05º57'N, 11º13'E | 15 | 50.0 | 13.3 | 36.7 | 1 | 2 | 0.067 | 0.00015 |
| Equatorial  Guinea | Total | 15 | 36.7 | 33.3 | 30.0 | 2 | 3 | 0.504 | 0.00120 |
|  | Miyobo (2003)  01°45'N, 10°10'E | 7 | 57.1 | 21.4 | 21.4 |  |  |  |  |
|  | Ngonamanga (2004)  02°08'N, 09°46'E | 8 | 18.8 | 43.8 | 37.5 |  |  |  |  |
| Bioko island | Sacriba (2003)  03°42'N, 08°43'E | 7 | 92.9 | 0.0 | 7.1 | 3 | 4 | 0.648 | 0.00176 |
| Gabon | Total | 72 | 47.9 | 27.8 | 24.3 | 5 | 6 | 0.464 | 0.00112 |
|  | Bakoumba (2000)  01º49'S, 13º01'E | 22 | 45.5 | 25.0 | 29.5 |  |  |  |  |
|  | Benguia (1999)  01°37'S, 13°26'E | 17 | 76.5 | 17.6 | 5.9 |  |  |  |  |
|  | Dienga (1999)  01°52'S, 12°40'E | 14 | 82.1 | 10.7 | 7.1 |  |  |  |  |
|  | Libreville (2000)  00°22'N, 09°26'E | 19 | 0.0 | 52.6 | 47.4 |  |  |  |  |
| Angola | Cabinda (2000)  05°32'S, 12°11'E | 18 | 36.1 | 41.7 | 22.2 | 2 | 3 | 0.256 | 0.00061 |

**Table S1 (continued)**

| Country | Locality (year of collection)  Latitude/Longitude | *N* ^a^ | *kdr* alleles ^b^ (%) | | | Intron-1 polymorphism | | | |
| --- | --- | --- | --- | --- | --- | --- | --- | --- | --- |
|  |  |  | 1014 L | 1014S | 1014F | *S* ^c^ | *h* ^d^ | *Hd* ^e^ | *π* ^f^ |
|  | | | | | | | | | |
| **East African region** | | | | | | | | | |
| Kenya | Asembo (2003)  00°10'S, 34°22'E | 13 | 38.5 | 61.5 | 0.0 | 0 | 1 | - | - |
| Tanzania | Ifakara (1999)  08°07'S, 36°40'E | 15 | 100.0 | 0.0 | 0.0 | 3 | 4 | 0.513 | 0.00129 |
| Malawi | Total | 13 | 100.0 | 0.0 | 0.0 | 2 | 3 | 0.557 | 0.00142 |
|  | Chipula (2003)  12°56'S, 34°17'E | 4 | 100.0 | 0.0 | 0.0 |  |  |  |  |
|  | Thyolo (1997)  16°04'S, 35°07'E | 9 | 100.0 | 0.0 | 0.0 |  |  |  |  |
| Mozambique | Furvela (2005)  23°43'S, 35°18'E | 19 | 100.0 | 0.0 | 0.0 | 2 | 3 | 0.494 | 0.00120 |
| Total |  | 288 | 59.4 | 14.6 | 26.0 | 8 | 9 | 0.323 | 0.00078 |

^a^ number of individuals analyzed.

^b^ 1014L: wild-type allele corresponding to a Leucine (TTA) residue at position 1014 of the *kdr* locus. 1014S: *kdr* allele encoding for a Serine, resulting from the Leucine (TTA)-Serine (TCA) substitution. 1014F: *kdr* allele encoding for Phenylalanine, resulting from the Leucine (TTA)-Phenylalanine (TTT) substitution.

^c^ number of segregating (polymorphic) sites.

^d^ number of different haplotypes

^e^ haplotype (gene) diversity.

^f^ nucleotide diversity (average number of nucleotide differences per site between two sequences).

* part of the intron-1 sequences for these sites have been included in a previous study [10], accounting for 44 (15.3%) out of the 288 individuals analyzed.
